# Efficacy and safety of acoziborole in patients with human African trypanosomiasis caused by *Trypanosoma brucei gambiense*: a multicentre, open-label, single-arm, phase 2/3 trial

**DOI:** 10.1016/S1473-3099(22)00660-0

**Published:** 2023-04

**Authors:** Victor Kande Betu Kumeso, Wilfried Mutombo Kalonji, Sandra Rembry, Olaf Valverde Mordt, Digas Ngolo Tete, Adeline Prêtre, Sophie Delhomme, Médard Ilunga Wa Kyhi, Mamadou Camara, Julie Catusse, Stefan Schneitter, Morgane Nusbaumer, Erick Mwamba Miaka, Hélène Mahenzi Mbembo, Joseph Makaya Mayawula, Mariame Layba Camara, Félix Akwaso Massa, Lewis Kaninda Badibabi, Augustin Kasongo Bonama, Papy Kavunga Lukula, Sylvain Mutanda Kalonji, Phyll Mariero Philemon, Ricardo Mokilifi Nganyonyi, Hugues Embana Mankiara, André Asuka Akongo Nguba, Vincent Kobo Muanza, Ernest Mulenge Nasandhel, Aimée Fifi Nzeza Bambuwu, Bruno Scherrer, Nathalie Strub-Wourgaft, Antoine Tarral

**Affiliations:** a Ministry of Health, Kinshasa, Democratic Republic of the Congo; b Drugs for Neglected Diseases initiative, Kinshasa, Democratic Republic of the Congo; c Drugs for Neglected Diseases initiative, Geneva, Switzerland; d Dipumba Hospital (MIBA), Mbuji-Mayi, Democratic Republic of the Congo; e Programme National de Lutte contre la Trypanosomiase Humaine Africaine, Conakry, Guinea; f Swiss Tropical and Public Health Institute, Allschwil, Switzerland; g University of Basel, Basel, Switzerland; h Programme National de Lutte contre la Trypanosomiase Humaine Africaine, Kinshasa, Democratic Republic of the Congo; i Bandundu Hospital, Vanga, Democratic Republic of the Congo; j HAT treatment centre, Dubreka, Guinea; k Masi Manimba Hospital, Masi-Manimba, Democratic Republic of the Congo; l Katanda Hospital, Mbuji-Mayi, Democratic Republic of the Congo; m Isangi Hospital, Isangi, Democratic Republic of the Congo; n Bagata Hospital, Bagata, Democratic Republic of the Congo; o Ngandajika Hospital, Kasaï Oriental, Democratic Republic of the Congo; p Kwamouth Hospital, Kwamouth, Democratic Republic of the Congo; q Bolobo Hospital, Bolobo, Democratic Republic of the Congo; r Roi Baudoin Hospital, Kinshasa, Democratic Republic of the Congo; s Nkara Hospital, Nkara, Democratic Republic of the Congo; t Kimpese Hospital, Kimpese, Democratic Republic of the Congo; u Bruno Scherrer Conseil, Saint Arnoult en Yvelines, France

## Abstract

**Background:**

Human African trypanosomiasis caused by *Trypanosoma brucei gambiense* (gambiense HAT) in patients with late-stage disease requires hospital admission to receive nifurtimox–eflornithine combination therapy (NECT). Fexinidazole, the latest treatment that has been recommended by WHO, also requires systematic admission to hospital, which is problematic in areas with few health-care resources. We aim to assess the safety and efficacy of acoziborole in adult and adolescent patients with gambiense HAT.

**Methods:**

This multicentre, prospective, open-label, single-arm, phase 2/3 study recruited patients aged 15 years or older with confirmed gambiense HAT infection from ten hospitals in the Democratic Republic of the Congo and Guinea. Inclusion criteria included a Karnofsky score greater than 50, ability to swallow tablets, a permanent address or traceability, ability to comply with follow-up visits and study requirements, and agreement to hospital admission during treatment. Oral acoziborole was administered as a single 960 mg dose (3 × 320 mg tablets) to fasted patients. Patients were observed in hospital until day 15 after treatment administration then for 18 months as outpatients with visits at 3, 6, 12, and 18 months. The primary efficacy endpoint was the success rate of acoziborole treatment at 18 months in patients with late-stage gambiense HAT (modified intention-to-treat [mITT] population), based on modified WHO criteria. A complementary post-hoc analysis comparing the 18-month success rates for acoziborole and NECT (using historical data) was performed. This study is registered at ClinicalTrials.gov, NCT03087955.

**Findings:**

Between Oct 11, 2016, and March 25, 2019, 260 patients were screened, of whom 52 were ineligible and 208 were enrolled (167 with late-stage and 41 with early-stage or intermediate-stage gambiense HAT; primary efficacy analysis set). All 41 (100%) patients with early-stage or intermediate-stage and 160 (96%) of 167 with late-stage disease completed the last 18-month follow-up visit. The mean age of participants was 34·0 years (SD 12·4), including 117 (56%) men and 91 (44%) women. Treatment success rate at 18 months was 95·2% (95% CI 91·2–97·7) reached in 159 of 167 patients with late-stage gambiense HAT (mITT population) and 98·1% (95·1–99·5) reached in 159 of 162 patients (evaluable population). Overall, 155 (75%) of 208 patients had 600 treatment-emergent adverse events. A total of 38 drug-related treatment-emergent adverse events occurred in 29 (14%) patients; all were mild or moderate and most common were pyrexia and asthenia. Four deaths occurred during the study; none were considered treatment related. The post-hoc analysis showed similar results to the estimated historical success rate for NECT of 94%.

**Interpretation:**

Given the high efficacy and favourable safety profile, acoziborole holds promise in the efforts to reach the WHO goal of interrupting HAT transmission by 2030.

**Funding:**

Bill & Melinda Gates Foundation, UK Aid, Federal Ministry of Education and Research, Swiss Agency for Development and Cooperation, Médecins Sans Frontières, Dutch Ministry of Foreign Affairs, Norwegian Agency for Development Cooperation, Norwegian Ministry of Foreign Affairs, the Stavros Niarchos Foundation, Spanish Agency for International Development Cooperation, and the Banco Bilbao Vizcaya Argentaria Foundation.

**Translation:**

For the French translation of the abstract see Supplementary Materials section.

## Introduction

Human African trypanosomiasis (HAT; also known as sleeping sickness) is caused by the protozoan parasite *Trypanosoma brucei gambiense* (gambiense HAT) and is transmitted by the tsetse fly. This neglected tropical disease is mostly fatal when left untreated.^1^, ^2^, ^3^ HAT remains endemic in sub-Saharan Africa, mainly in the Democratic Republic of the Congo, although surveillance and control programmes have achieved a steady decline in gambiense HAT incidence worldwide with 565 cases reported in 2020 (a decrease from 2110 cases in 2016).^4^

**Research in context**

**Evidence before this study**

The choice of treatment for human African trypanosomiasis (HAT) has traditionally depended on the stage of disease. Once parasites proliferate beyond the haemolymphatic system (early-stage disease) and invade the CNS, drugs need to cross the blood–brain barrier to treat neuroencephalitic infection (late-stage disease). Until 2019, patients with early-stage gambiense HAT were given intramuscular pentamidine injections for 7 days, whereas those with late-stage disease received nifurtimox–eflornithine combination therapy (NECT), which requires complex logistics and needs to be administered in hospital. Treatment of gambiense HAT has improved with the development of fexinidazole, the only oral drug available for late-stage disease, and despite its substantial advantages over NECT, disease severity still needs to be assessed (possibly involving a lumbar puncture) and treatment should be taken for 10 days. We searched PubMed for articles published from Sept 25, 2017, to Sept 25, 2022, using the search terms “human African trypanosomiasis”, “treatment”, “NECT”, and “fexinidazole”, with no language restrictions. A disease-stage independent treatment, which would be simpler to administer and as efficacious as NECT, is still needed. To fulfil this unmet need, the Drugs for Neglected Diseases initiative (in collaboration with Anacor Pharmaceuticals and Scynexis) has discovered acoziborole, a new orally active compound with preclinical activity against gambiense HAT parasites, good tolerability, and which reaches the required exposure to the parasite in blood and cerebrospinal fluid after a single dose. This multicentre, prospective, phase 2/3 study was designed to assess the safety and efficacy of a single 960 mg oral dose of acoziborole administered to patients in the fasting state with gambiense HAT.

**Added value of this study**

We showed a positive benefit–risk profile for using a single oral dose of acoziborole to treat gambiense HAT in adults and adolescents, regardless of disease stage. No substantial drug-related safety signals were identified and the primary objective was met, with 159 (95% [95% CI 91·2–97·7]) of 167 patients who had late-stage gambiense HAT in the intention-to-treat population reaching treatment success at 18 months, which was similar to the estimated historical results for NECT with 354 (94% [Jeffreys 95% CI 91·4–96·2]) of 376 patients. In the early-stage and intermediate-stage cohort, all 41 (100%) patients reached treatment success at 18 months. A single oral dose of acoziborole is a clear improvement compared with 10 days of NECT since disease staging by painful lumbar puncture and hospital admission for intravenous treatment are not required, and the treatment duration is shorter.

**Implications of all the available evidence**

Due to the substantial decline in incidence, enrolling patients with gambiense HAT into clinical trials is challenging. Following advice from the European Medicines Agency, this study was designed as an open-label, single-arm trial with no comparator or control group. Given high treatment success rates and good safety results in this study, the WHO HAT elimination Technical Advisory Group recommended that a double-blind trial should be conducted to investigate the use of acoziborole versus placebo in seropositive patients with negative parasitological assays to generate further safety data, with an aim of contributing to the WHO target of interrupting HAT transmission by 2030. This randomised study is ongoing (NCT05256017), with 900 participants in the acoziborole group versus 300 in the placebo group.

There are two clinical stages of gambiense HAT. In early-stage (haemolymphatic) gambiense HAT, trypanosomes are present in the blood and lymphatic system and symptoms remain non-specific (eg, intermittent fever, headache, joint pain, pruritus, and lymphadenopathy).^1^ Without treatment, HAT progresses to late-stage (meningoencephalitic) disease with trypanosomes crossing the blood–brain barrier and development of neurological symptoms (eg, mental confusion, behavioural changes, and sensory and sleep disturbances), which eventually lead to coma and death.^1^, ^5^

Therapeutic options depend on disease stage. Patients with early-stage gambiense HAT often receive pentamidine (usually as an intramuscular injection once per day for 7–10 days)^6^ and those with late-stage disease require hospital admission and receive nifurtimox–eflornithine combination therapy (NECT; oral nifurtimox three times per day for 10 days and intravenous eflornithine two times per day for 7 days).^6^, ^7^, ^8^, ^9^ The new WHO guidelines for treatment of gambiense HAT indicate the use of fexinidazole with some precautions and restrictions as a first-line treatment for patients aged 6 years or older with a bodyweight of 20 kg or higher.^10^

Acoziborole, an oral benzoxaborole-6-carboxamide from the Drugs for Neglected Diseases initiative (DNDi) drug discovery programme, showed high activity in vitro in both stages of gambiense HAT in murine models,^11^ and low toxicity and strong efficacy in preclinical studies allowing progression to phase 1.^12^ A first-in-human study (unpublished) showed the tolerability and pharmacokinetic profile of acoziborole and determined that a single therapeutic dose of 960 mg in fasted patients provided the desired exposure to the parasite in blood and cerebrospinal fluid (CSF) with a good safety profile. Here, we aim to assess the safety and efficacy of acoziborole in adult and adolescent patients with gambiense HAT.

## Methods

### Study design and participants

This multicentre, prospective, open-label, single-arm, phase 2/3 study recruited patients aged 15 years or older with confirmed gambiense HAT infection from ten hospitals in the Democratic Republic of the Congo and Guinea. Late-stage gambiense HAT diagnosis required presence of trypanosomes in the blood, lymph, or CSF (or a combination of either) and more than 20 cells per μL in CSF. Early-stage and intermediate-stage gambiense HAT required the presence of trypanosomes in the blood or lymph (or both), but absence of trypanosomes in the CSF and a CSF white blood cell (WBC) count of 6–20 cells per μL (intermediate stage) or 5 cells per μL or fewer (early stage). Inclusion criteria included a Karnofsky score greater than 50, ability to swallow tablets, a permanent address or traceability, ability to comply with follow-up visits and study requirements, and agreement to hospital admission during treatment. Exclusion criteria were severe malnutrition (BMI <16 kg/m^2^), pregnancy or breastfeeding, any clinically significant medical condition that could jeopardise patient safety or study participation, previous treatment for HAT (except pentamidine), previous enrolment in the study, and current alcohol or drug misuse. All participants provided written informed consent; for patients younger than 18 years assent was provided by a parent or legal presentative and for illiterate patients, consent was provided by a witness. This study was designed and performed in accordance with the Declaration of Helsinki and International Council for Harmonisation E6 Good Clinical Practice Guidelines. The study protocol^13^ was approved by the subregional Ethics Committee for Health Research in Central Africa and the national ethics committees in the Democratic Republic of the Congo and Guinea.

### Procedures

Oral acoziborole was administered as a single 960 mg dose (3 × 320 mg tablets) to fasted patients (for at least 2 h before dosing). The study started with a pretreatment period of up to 15 days with baseline assessments, malaria testing, antimalarials (as needed), and anthelmintics given at least 3 days before acoziborole. Patients were observed in hospital until day 15 after treatment administration then for 18 months as outpatients with visits at approximately 3, 6, 12, and 18 months. During hospital admission, signs and symptoms of HAT were evaluated; neurological and physical examinations were performed; vital signs were recorded; and blood samples for haematology (haemoglobin, complete WBC count, and platelets), biochemistry (albumin, alkaline phosphatase, alanine aminotransferase, aspartate aminotransferase, blood urea nitrogen, chloride, creatinine, glucose, potassium, sodium, calcium, total bilirubin, bicarbonate, and total protein), and thyroid function were taken at baseline, day 5 and 11, and at each follow-up visit. Electrocardiograms (ECGs) and blood sampling for pharmacokinetic analysis were performed at baseline and at 4, 9, 24, 48, 72, 96, and 240 h after dosing. A concentration–response analysis was performed to assess the relationship between pharmacokinetics and QT interval with Fredericia (QTcFri) of acoziborole in patients with valid ECG that matched pharmacokinetic sampling. Pregnancy tests were done at baseline, day 11, and at each visit. Adverse events and concomitant medications were recorded during hospital stay until 6 months and serious adverse events were monitored until 18 months. Assessments including blood, lymph, and CSF sampling for the detection of trypanosomes and CSF WBC count (lumbar puncture) were performed at baseline, day 11, and follow-up visits (6, 12, and 18 months; appendix 2 p 2). The presence of trypanosomes was determined by direct microscopy using the mini anion exchange centrifugation technique and recorded by video. Photographs were taken of the Fuchs-Rosenthal counting chamber (LO-Laboroptik, Lancing, UK) used for WBC count. Blood samples were also collected for trypanosome spliced leader RNA testing for the DiTECT-HAT-WP4 study.^14^ Patients who relapsed received NECT as rescue treatment and were referred to the national HAT control programme.

### Outcomes

The primary efficacy endpoint was the treatment outcome (success or failure) in patients with late-stage HAT assessed 18 months after acoziborole treatment (per protocol population), based on modified WHO criteria.^15^ At 18 months, success was defined as cure or probable cure (absence of trypanosomes and <20 WBC per μL of CSF). Failure was defined as relapse, probable relapse, death from any cause, use of rescue medication, loss to follow-up, refusal of post-treatment lumbar puncture, and—in the absence of a lumbar puncture at 18 months—an unfavourable outcome before 18 months or signs and symptoms evoking a relapse at 18 months (appendix 2 p 10). Secondary endpoints were success rate at 12 months (appendix 2 p 12) and time to treatment failure in patients with late-stage gambiense HAT. Secondary efficacy endpoints were treatment outcomes (success or failure) at 6, 12, and 18 months in all patients (including those with early-stage HAT). Safety endpoints were monitored extensively and included adverse events (graded using National Cancer Institute Common Terminology Criteria for Adverse Events; version 4.03), HAT signs and symptoms, neurological and physical examinations, vital signs, standard haematology blood chemistry, and thyroid hormones (appendix 2 p 4).

A complementary post-hoc analysis of the 18-month success rate was done to compare acoziborole (the primary analysis) with NECT (using data from Priotto and colleagues^12^ and Mesu and colleagues^13^) and fexinidazole (using data from Mesu and colleagues^13^). Additionally, the propensity score was calculated using baseline data (WBC in CSF, age, and sex).

### Statistical analysis

Based on the maximum feasible enrolment in a reasonable timeframe of 2 years, the sample size was fixed to 162 patients with late-stage gambiense HAT, including an expectation of seven dropouts (5%), but we did not predetermine the number of patients with early-stage or intermediate-stage gambiense HAT. A first cohort of patients with late-stage gambiense HAT was recruited and after futility analyses and study data review by the data safety monitoring board for safety and tolerability, a second cohort of patients with early-stage and intermediate-stage disease was recruited after 8 months.

The modified intention-to-treat (mITT; defined as all patients who received treatment, excluding those who fled the region due to armed conflict, a natural disaster, or force majeure and for whom no treatment failure was detected early and no data were available at 12 and 18 months) and per protocol populations (defined as all mITT patients with no major protocol deviations) were identical (appendix 2 p 5). The primary efficacy analysis was performed in the mITT population of patients with late-stage gambiense HAT. An estimate of the success rate at 18 months and Jeffreys 95% CIs were provided. Prespecified sensitivity analyses and post-hoc complementary analyses were also performed for the primary efficacy endpoint in the mITT (appendix 2 p 5). Success rates at 6 and 12 months in patients with late-stage gambiense HAT; at 6, 12, and 18 months in patients with early-stage or intermediate-stage disease; and at 18 months in all patients, were estimated as described for the primary endpoint. Time to proven and definitive failure was analysed according to cumulative failure rate using the Kaplan-Meier approach. Safety analyses were performed in the treated population, defined as all patients who received at least one tablet of acoziborole. Efficacy analysis (success rate) was performed in the evaluable population, defined as all mITT patients (excluding those who were lost to follow-up [except if a patient already had treatment failure]; had no post-treatment lumbar puncture [but with no treatment failure]; died for reasons unrelated to efficacy, safety, or disease progression; or withdrew consent before the 6-month visit). Comparisons with the yardstick were performed with JMP (version 7.0; SAS institute, Cary, NC, USA), STATA, and Cytel StatXact (StatXAct 9, Cytel, Waltham, MA, USA). All summaries and statistical analyses were generated using SAS (version 9.4). For population pharmacokinetic analysis, a one-compartment model was used with a sequential mixture of first-order absorption followed by a zero-order absorption and linear elimination. The derived parameters (HAT stage, gender, concomitant medications, and treatment-emergent adverse events) were plotted on a graph. This study is registered with ClinicalTrials.gov, NCT03087955.

### Role of the funding source

The funders of the study had no role in study design, data collection, data analysis, data interpretation, or writing of the report.

## Results

Between Oct 11, 2016, and March 25, 2019, 260 patients were screened, of whom 52 were ineligible and 208 were enrolled (167 with late-stage and 41 with early-stage or intermediate-stage gambiense HAT; figure). All 41 (100%) patients with early-stage or intermediate-stage and 160 (96%) of 167 with late-stage disease completed the last 18-month follow-up visit. The mean age of participants was 34·0 years (SD 12·4), including 117 (56%) men and 91 (44%) women. 174 (84%) of 208 patients were recruited in the Democratic Republic of the Congo and 34 (16%) in Guinea. Inclusion of this study in ClinicalTrials.gov was delayed due to an operational issue. Corrective actions were taken by the funder to prevent recurrence of this issue.

**Figure:**
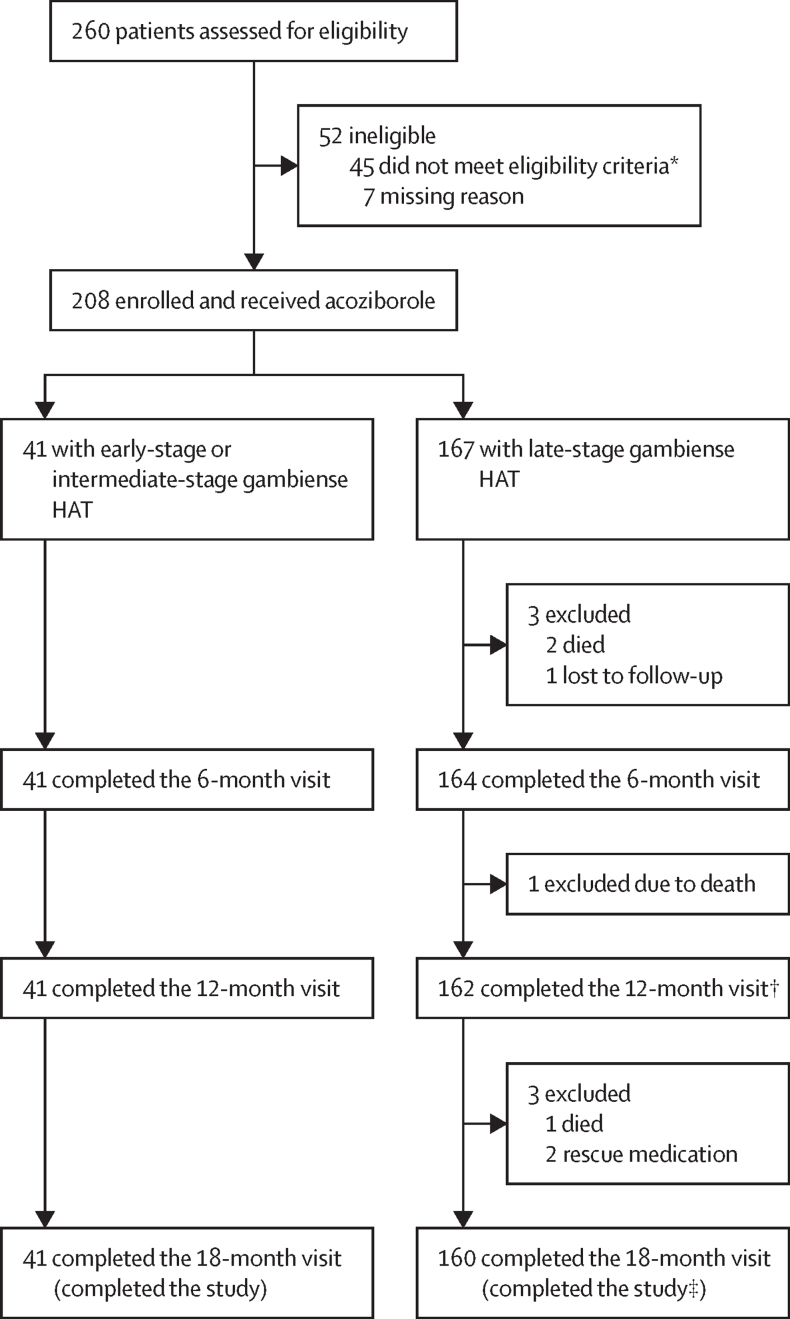
Patient disposition Gambiense HAT refers to HAT caused by *Trypanosoma brucei gambiense*. HAT=human African trypanosomiasis. *Including one patient who died during screening. †One patient with late-stage gambiense HAT missed the 12-month visit but attended the 18-month visit. ‡One patient who completed the study received rescue medication after the 18-month visit.

Trypanosomes in the CSF were detected in 139 (83%) of 167 patients with late-stage disease and 128 (77%) of those had more than 100 WBC per μl of CSF (table 1). The overall mean WBC count in CSF at baseline was 398·8 cells per μl (SD 438·4; table 2). A rapid decline of mean WBC count per μl of CSF was seen in the mITT population during follow-up visits. In the early-stage and intermediate-stage cohort, all 41 (100%) patients reached treatment success at all timepoints. Medical history and clinical HAT presentation were consistent with disease stage and clinical signs and symptoms were reported more often in patients with late-stage versus early-stage or intermediate-stage gambiense HAT at baseline (eg, drowsiness 124 [74%] of 167 *vs* six [15%] of 41, headache 107 [64%] *vs* 23 [56%], asthenia 96 [58%] *vs* 11 [27%], pruritus 96 [58%] *vs* 11 [27%], fever 75 [45%] *vs* 18 [44%], insomnia 67 [40%] *vs* ten [24%], and weight loss 67 [40%] *vs* ten [24%]).

**Table 1:** Baseline characteristics (modified intention-to-treat population)

	**Early stage or intermediate stage (n=41)**	**Late stage (n=167)**	**Overall (n=208)**
**Demographics**			
Men	15 (37%)	102 (61%)	117 (56%)
Women	26 (63%)	65 (39%)	91 (44%)
Age, years	40·9 (15·0)	32·4 (11·2)	34·0 (12·4)
Weight, kg	52·2 (9·2)	53·4 (10·5)	53·2 (10·2)
BMI, kg/m^2^	20·2 (2·7)	20·0 (2·6)	20·0 (2·6)
Parasitological findings*			
Blood positive	41 (100%)	165 (99%)	206 (99%)
Lymph node positive	17 (41%)	64 (38%)	81 (39%)
Cerebrospinal fluid positive	0	139 (83%)	139 (67%)
White blood cells >100 cells per μL	0	128 (77%)	128 (62%)

Data are presented as n (%) or mean (SD).

* Documented with video.

**Table 2:** WBC count per μL of cerebrospinal fluid from baseline to 18 months follow-up in the modified intention-to-treat population

	**Early stage or intermediate stage**	**Late stage**	**Overall**
**WBC at baseline**			
Number of patients	41	166	207
Mean	7·8 (5·3)	398·8 (438·4)	321·3 (422·3)
Median	6·0 (4·0–11·0)	260·0 (114·0–530·0)	190·0 (24·0–246·0)
**WBC at 11 days**			
Number of patients	41	167	208
Mean	4·9 (3·4)	106·7 (139·0)	86·6 (130·9)
Median	4·0 (3·0–6·0)	62·0 (26·0–124·0)	42·5 (10·0–102·5)
**WBC at 6 months**			
Number of patients	41	163	204
Mean	3·4 (2·8)	12·8 (13·1)	10·9 (12·3)
Median	3·0 (2·0–4·0)	9·0 (4·0–16·0)	7·0 (3·0–15·0)
**WBC at 12 months**			
Number of patients	41	161	202
Mean	2·0 (1·6)	6·8 (13·6)	5·8 (12·3)
Median	2·0 (1·0–3·0)	4·0 (3·0–7·0)	3·0 (2·0–6·0)
**WBC at 18 months**			
Number of patients	41	159	200
Mean	1·6 (1·3)	4·5 (20·1)	3·9 (18·0)
Median	1·0 (1·0–2·0)	2·0 (1·0–4·0)	2·0 (1·0–4·0)

Data are n, mean (SD), or median (IQR). WBC=white blood cells.

In terms of the primary efficacy endpoint, treatment success rate at 18 months was 95·2% (95% CI 91·2–97·7) reached in 159 of 167 patients with late-stage gambiense HAT (mITT population) and 98·1% (95·1–99·5) reached in 159 of 162 patients (evaluable population). For secondary efficacy endpoints, the treatment success rates at 6, 12, and 18 months were similar (appendix 2 p 6) and compare favourably with rates observed in historical NECT studies in a post-hoc analysis (estimated at 94·1% [91·4–96·2] reached in 354 of 376 patients; appendix 2 pp 6, 15). At 18 months, treatment failure occurred in eight patients with late-stage HAT (4·8% [2·21–9·05]; three relapses after receiving rescue therapy; four deaths unrelated to efficacy, safety, or disease evolution; and one loss to follow-up). The evaluable population (closely aligned with WHO criteria) excluded five (63%) of those eight patients from the mITT analysis. Kaplan-Meier analysis showed that the cumulative failure rate at 18 months in those with late-stage disease was 0·96 (95% CI 0·91–0·98; appendix 2 p 16).

Complementary post-hoc analysis after adjustment for key baseline characteristics (because patients in pivotal NECT had less severe disease than in our study) showed that the success rates of acoziborole and NECT were similar regardless of disease severity (based on CSF WBC count; appendix 2 pp 8, 15).^9^, ^16^, ^17^

The overall frequency of all signs and symptoms of HAT decreased during the study from day 15, reaching a low frequency or absence at 18 months (appendix 2 pp 9, 14). Overall, 155 (75%) of 208 patients in the treated population had 600 treatment-emergent adverse events; of those, 556 (93%) were mild or moderate, 44 (7%) were severe, and 27 (5%) were serious (table 3). The most frequently reported treatment-emergent adverse events were procedural pain (52 [25%]) and procedural headache (32 [15%]), both related to lumbar puncture; headache (51 [25%]); pyrexia (31 [15%]); and malaria (29 [14%]; appendix 2 p 7). A total of 38 drug-related treatment-emergent adverse events occurred in 29 (14%) patients; all were mild or moderate in intensity and were reported on days 1–5. Pyrexia (ten [5%]) and asthenia (six [3%]) were the most frequently reported drug related treatment-emergent adverse events (appendix 2 p 9).

**Table 3:** Summary of all adverse events in the treated population

	**Early stage or intermediate stage (n=41)**	**Number of events**	**Late stage (n=167)**	**Number of events**	**Overall (n=208)**	**Number of events**
At least one adverse event	33 (80%)	129	136 (81%)	613	169 (81%)	742
At least one TEAE	28 (68%)	99	127 (76%)	501	155 (75%)	600
At least one mild or moderate TEAE	27 (66%)	94	127 (76%)	462	154 (74%)	556
At least one severe TEAE	4 (10%)	5	24 (14%)	39	28 (14%)	44
At least one drug-related TEAE	2 (5%)	2	27 (16%)	36	29 (14%)	38
At least one severe drug-related TEAE	0	0	0	0	0	0
At least one serious TEAE	3 (7%)	4	18 (11%)	23	21 (10%)	27
At least one serious drug-related TEAE	0	0	0	0	0	0
At least one serious TEAE leading to death	0	0	4 (2%)	4	4 (2%)	4

Data are presented as n (%), unless stated otherwise. TEAE=treatment-emergent adverse event.

Serious treatment-emergent adverse events were reported in 21 (10%) of 208 patients, but none of these events were considered drug-related (appendix 2 p 8). Infections and infestations occurred in nine (4%) patients. Psychiatric disorders occurred only in patients with late-stage gambiense HAT (six [3%]); four events in three of these patients occurred 3 months after treatment and although psychiatric signs and symptoms improved during hospital stay, follow-up psychiatric consultation was refused when proposed by the investigators (MIWK, AKB, and VKM). The investigator and data safety monitoring board considered these events were possible sequelae of late-stage gambiense HAT, in line with previous reports (appendix 2 p 8).^18^, ^19^

During the study, incidental pregnancies were detected within 6 months of acoziborole treatment in seven women; in utero exposure was considered in six of these women, all of which were full-term pregnancies without complications. Four children were born without health problems (pregnancies diagnosed on days 11, 30, 85, and 140 after dosing), and three newborn babies died (death at delivery due to cord prolapse and no assistance for 10 h; acute neonatal infection; and accidental suffocation during breastfeeding). Five further pregnancies and deliveries were observed outside of the reporting period (>6 months after drug intake) without any health complications.

Four deaths occurred after dosing in the treated population (poisoning on day 24, worsening of progressive peripheral ascending polyneuropathy on day 9, extrapulmonary tuberculosis on day 233, and acute pulmonary oedema on day 387) and none were considered related to treatment or HAT. The laboratory results showed no substantial changes from baseline in haematology parameters, liver enzymes (alanine and aspartate aminotransferases), and other biochemistry data. Thyroid function tests (thyroid-stimulating hormone [TSH], free tri-iodothyronine [T_3_], and free thyroxine [T_4_]) showed no clinically significant abnormal values. Light fluctuations of TSH in the upper limit of normal appeared on day 11 and normalised 3 months after dosing. There were no TSH results higher than 10 IU/mL (the threshold for treatment).^20^ ECG recordings with central re-readings detected no out-of-range values. The corrected QTcFri formulae detected no significant effect of acoziborole on QTcFri. The estimated difference in QTcFri at the highest geometric mean exposure of acoziborole after a 960 mg dose was estimated at –9·3 ms (90% CI –10·1 to –8·4).

Pharmacokinetic analysis revealed a T_max_ of 48 h and elimination T_1/2_ of 229·78 h. The area under the curve (AUC)_0–inf_ was estimated at 4256·11 (μg × h per mL). Overall, no difference in exposure was observed for HAT stage, gender, or concomitant medications, and there was no relationship between exposure and severity of treatment-emergent adverse events. The planned logistical regression between exposure and efficacy was not feasible due to low treatment failure (three [1%] of 208 patients). However, a deeper investigation was conducted in patients with confirmed treatment failure; their exposures were compared with the overall population and demographic characteristics, laboratory values (aspartate aminotransferase, alanine aminotransferase, alkaline phosphatase, total bilirubin, total protein, and albumin), and treatment adherence were investigated. Overall, no clear common explanation was found for these three patients, although an absence of efficacy linked to low exposure could not be entirely excluded for the two first patients.

## Discussion

In this open-label, non-comparative study, a single 960 mg oral dose of acoziborole in fasted conditions showed an efficacy of 95·2% reached in 159 of 167 patients with late-stage gambiense HAT, corresponding to results from studies with NECT (94%).^16^, ^17^ The success rate at 18 months in the evaluable population, which excluded four deaths for reasons not linked to disease or study treatment, was 98·1% reached in 159 of 162 patients. The main difference between these findings and previous studies was that microscopic detection of the parasite was documented by video recording and pictures were taken of CSF WBC in a Fuchs-Rosenthal counting chamber during screening. These methods were performed at each follow-up visit throughout the whole study in addition to multiple criteria (clinical, parasitological, ECG, and biological) used to evaluate the outcome. The patients were mostly recruited at hospitals when consulting for symptoms (passive screening), which might explain why patients with late-stage HAT had more severe disease than those in previous studies (83% were trypanosome positive in the CSF and 77% had >100 WBC per μl CSF).^3^ Finally, since missing post-treatment lumbar puncture, death due to any cause, and loss to follow-up were all considered as failure, the assessment of treatment success was stringent.

The criteria for success used in previous NECT studies were less stringent and consequently less favourable towards acoziborole. In addition to these differences, acoziborole is less cumbersome than previous treatments, which is an important advantage because the drug requires a single oral administration in fasted conditions compared with 10 days of oral fexinidazole administered with food or NECT administered in hospital (twice per day infusions of eflornithine for 7 days plus 10 days of three times per day of oral nifurtimox). The logistics (ie, shipment and drug storage) are simplified as acoziborole is packaged in a small box whereas NECT is packaged in a 4 kg box. Patient management is easier, unlike with NECT, because administration of acoziborole does not require hospital admission, highly skilled health personnel, or a lumbar puncture.

The principal limitation of this study was the absence of a comparator or control group, precluding randomisation; instead comparisons were made with historical data, as permitted by WHO.^15^ The sample size was discussed with the European Medicines Agency (July 26, 2018) and was based on the maximum feasible enrolment within a reasonable timeframe, because of the rapid decline in HAT incidence and to expose the test drug to as many patients as possible. As suggested by the European Medicines Agency, adjustments for baseline characteristics were made in the complementary analysis comparing the success rate of acoziborole with NECT in three previous studies.^9^, ^16^, ^17^

The proportion of treatment-emergent adverse events related to treatment was low and all events were mild or moderate in intensity. No substantial drug-related safety signals were identified in this study. All 38 drug-related treatment-emergent adverse events occurred on days 1–5, mainly on day 1. Serious events were reported more often in patients with late-stage gambiense HAT and mainly occurred after patients were discharged from hospital; none were considered treatment related. The safety profile of acoziborole compares favourably with those of NECT and pentamidine.^9^, ^12^, ^16^, ^17^, ^21^, ^22^ The incidental pregnancies that occurred after dosing had no complications, which is in line with preclinical data (unpublished). The main difficulties occurred during delivery, as the women gave birth without assistance, leading to death of three newborn babies. In future studies, the time of delivery should be carefully monitored since incidental pregnancies cannot be excluded.

Pharmacokinetic analysis was performed on dry blood spots and CSF sampling, and supported the results observed in healthy volunteers in a phase 1 study (unpublished). The long half-life (around 14 days) and exposure observed in blood and CSF mean that concentrations of the active drug remain higher than the antiparasitic efficacy (EC_90_) against *T b gambiense* for at least 2 months after dosing.

Based on these findings, the benefit–risk profile of acoziborole for adults and adolescents with gambiense HAT, regardless of disease stage, is considered positive. Given the high efficacy and good safety at all stages of disease, acoziborole eliminates the need for routine lumbar puncture at diagnosis and during follow-up, which requires trained staff, is associated with complications, and is a source of anxiety for patients. These advantages and the fact that a single oral dose would be more accessible to patients living in remote areas without easy access to health care, means that acoziborole holds promise in the efforts to reach the WHO goal of interrupting HAT transmission by 2030. To facilitate the use of acoziborole in treatment of suspected patients with negative parasitological assays for gambiense HAT, the integration of new tools into the national and global policies part of WHO HAT elimination Technical Advisory Group has recommended extending the safety database to detect uncommon adverse events. A double-blind safety study investigating the use of acoziborole versus placebo in this at-risk population is ongoing in the Democratic Republic of the Congo and Guinea (NCT05256017).

**This online publication has been corrected. The corrected version first appeared at thelancet.com/infection on December 19, 2022**

## Data sharing

The data underlying the results of this study are available upon request. Researchers can contact the DNDi for data access requests at CTdata@dndi.org. Researchers can also request data by completing the form available at https://www.dndi.org/category/clinical-trials/. The authors need to confirm that data and results will be shared with the DNDi and will be published under open access.

## Declaration of interests

BS reports fees from the Drugs for Neglected Diseases initiative (DNDi) for the statistical report and consulting fees from CEMAG, D&A Pharma, Inventiva, and OT4B Pharma. WMK, SR, AP, OVM, NS-W, DNT, and AT report employment at the DNDi. SS declares that Swiss Tropical and Public Health Institute acted as a service provider for the DNDi by monitoring the study sites. All other authors declare no competing interests.

## Supplementary Materials

French translation of the abstract
