# Correction to *Lancet Infect Dis* 2022; published online Nov 29. https://doi.org/10.1016/S1473-3099(22)00660-0

**DOI:** 10.1016/S1473-3099(22)00859-3

**Published:** 2023-02

*Kumeso VKB, Mutombo Kalonji W, Rembry S, et al. Efficacy and safety of acoziborole in patients with human African trypanosomiasis caused by Trypanosoma brucei gambiense: a multicentre, open-label, single-arm, phase 2/3 trial.* Lancet Infect Dis *2022; published online Nov 29. https://doi.org/10.1016/S1473-3099(22)00660-0*—In this Article, the inclusion criteria should have been “a Karnofsky score greater than 50” throughout the Article. This correction has been made to the online version as of Dec 19, 2022, and will be made to the printed version.

