# Supplementary material for: Efficacy and safety of acoziborole in patients with human African trypanosomiasis caused by Trypanosoma brucei gambiense: a multicentre, open-label, single-arm, phase 2/3 trial
Source: Lancet Infect Dis. 2023 Apr;23(4):463–70. doi: 10.1016/S1473-3099(22)00660-0 (PMC10033454; doi:10.1016/S1473-3099(22)00660-0)
Supplement: French translation of the abstract [file mmc1.pdf]

THE LANCET
Infectious Diseases

Supplementary appendix 1

This translation in French was submitted by the authors and we reproduce it as supplied. It has not been peer reviewed. *The Lancet*'s editorial processes have only been applied to the original in English, which should serve as reference for this manuscript.

Cette traduction en français a été proposée par les auteurs et nous l'avons reproduite telle quelle. Elle n'a pas été examinée par des pairs. Les processus éditoriaux du *Lancet* n'ont été appliqués qu'à l'original en anglais et c'est cette version qui doit servir de référence pour ce manuscrit.

Supplement to: Betu Kumeso VK, Mutombo Kalonji W, Rembry S, et al. Efficacy and safety of acoziborole in patients with human African trypanosomiasis caused by Trypanosoma brucei gambiense: a multicentre, open-label, single-arm, phase 2/3 trial. *Lancet Infect Dis* 2022; published online Nov 29. https://doi.org/10.1016/S1473-3099(22)00660-0.

Généralités Les patients à un stade avancé de la trypanosomiase humaine africaine (THA) due à *Trypanosoma brucei gambiense* (gTHA) () doivent être hospitalisés pour recevoir l'association thérapeutique nifurtimox-éflornithine (NECT). Le fexinidazole, dernier traitement recommandé par l'OMS, nécessite également une hospitalisation systématique, difficile à réaliser dans les régions disposant de peu de ressources sanitaires. Notre objectif était d'évaluer la sécurité et l'efficacité de l'acoziborole chez des patients adultes et adolescents atteints de gTHA

Méthodes Cette étude de phase 2/3, multicentrique, ouverte et à un seul bras a été réalisée dans dix hôpitaux en République démocratique du Congo et en Guinée chez des patients âgés de 15 ans ou plus et atteints de gTHA confirmée. Les critères d'inclusion comprenaient un score de Karnofsky inférieur à 50, la capacité d'avaler des comprimés, une adresse permanente ou une traçabilité, la capacité de se conformer aux visites de suivi et aux exigences de l'étude, ainsi que l'acceptation de l'hospitalisation pendant le traitement. L'acoziborole a été administré par voie orale aux patients à jeun, en une dose unique de 960 mg (3 comprimés de 320 mg). Les patients sont restés en observation à l'hôpital pendant 15 jours après la prise du traitement, et ils ont ensuite été suivi en ambulatoire pendant 18 mois, à 3, 6, 12 et 18 mois. Le critère d'évaluation principal de l'efficacité était le taux de succès du traitement par acoziborole à 18 mois chez des patients atteints de gTHA à un stade avancé (population en intention de traiter [ITT modifiée)), selon les critères modifiés de l'OMS. Une analyse post-hoc complémentaire a été réalisée pour comparer les taux de succès à 18 mois de l'acoziborole et de NECT (données historiques). Cette étude est enregistrée sur ClinicalTrials.gov, sous la référence NCT03087955.

Résultats Entre le 11 octobre 2016 et le 25 mars 2019, 260 patients ont été sélectionnés, parmi lesquels 52 étaient inéligibles et 208 ont été recrutés dans l'étude (167 atteints de gTHA à un stade avancé et 41 atteints de gTHA à un stade précoce ou intermédiaire ; ensemble de données d'efficacité primaires). Les 41 patients (100 %) atteints de la maladie à un stade précoce ou intermédiaire, et 160 des 167 patients (96 %) atteints de la maladie à un stade avancé ont effectué la dernière visite de suivi à 18 mois. L'âge moyen des participants était de 34,0 ans (écart-type 12,4), dont 117 hommes (56 %) et 91 femmes (44 %). Le taux de réussite du traitement à 18 mois était égal à 95,2% (IC à 95 % 91,2-97,7) chez 159 des 167 patients atteints de gTHA au stade avancé (population ITT modifiée), et à 98,1 % (95,1-99,5) chez 159 des 162 patients évaluables. Au total, 600 événements indésirables apparus sous traitement ont été rapportés chez 155 des 208 patients (75 %), et 38 événements indésirables apparus sous traitement et liés au médicament ont été rapportés chez 29 patients (14 %) ; tous étaient légers ou modérés, et les plus fréquents étaient fièvre et asthénie. Quatre décès sont survenus pendant l'étude, mais aucun n'a été considéré comme lié au traitement. L'analyse post-hoc a montré des résultats similaires au taux de réussite historique estimé de 94 % pour NECT.

Interprétation Compte tenu de son efficacité élevée et de son profil de sécurité favorable, l'acoziborole est un outil prometteur pour atteindre l'objectif de l'OMS d'interrompre la transmission de la THA d'ici 2030.

Financement Par l'intermédiaire de DND*i* (Drugs for Neglected Diseases *initiative*) : the Bill & Melinda Gates Foundation (INV002384 and INV-008203); UK Aid; the Federal Ministry of Education and Research (BMBF) through KfW, Germany; the Swiss Agency for Development and Cooperation (SDC); Médecins Sans Frontières; the Dutch Ministry of Foreign Affairs (DGIS), the Netherlands; the

Norwegian Agency for Development Cooperation (Norad), Norwegian Ministry of Foreign Affairs, as part of Norway's in-kind contribution to the second European & Developing Countries Clinical Trials Partnership programme (EDCTP2); the Stavros Niarchos Foundation; the Spanish Agency for International Development Cooperation (AECID), Spain; the BBVA Foundation (through the Frontiers of Knowledge Award in Development Cooperation).
