# Supplementary material for: Efficacy and safety of acoziborole in patients with human African trypanosomiasis caused by Trypanosoma brucei gambiense: a multicentre, open-label, single-arm, phase 2/3 trial
Source: Lancet Infect Dis. 2023 Apr;23(4):463–70. doi: 10.1016/S1473-3099(22)00660-0 (PMC10033454; doi:10.1016/S1473-3099(22)00660-0)
Supplement: Supplementary appendix 2 [file mmc2.pdf]

THE LANCET
Infectious Diseases

Supplementary appendix 2

This appendix formed part of the original submission and has been peer reviewed. We post it as supplied by the authors.

Supplement to: Betu Kumeso VK, Mutombo Kalonji W, Rembry S, et al. Efficacy and safety of acoziborole in patients with human African trypanosomiasis caused by Trypanosoma brucei gambiense: a multicentre, open-label, single-arm, phase 2/3 trial. *Lancet Infect Dis* 2022; published online Nov 29. https://doi.org/10.1016/S1473-3099(22)00660-0.

Supplementary Appendix

Table S1: Schedule of assessments **2**

Table S2: Study objectives **4**

Table S3: Criteria for evaluation **4**

Table S4: Analysis sets **5**

Table S5: Statistical methods **5**

Table S6: Historical data with NECT in late-stage g-HAT patients: determination of yardstick **6**

Table S7: Success rates at 18, 12 and 6 months (mITT set) **6**

Table S8: Summary of treatment-emergent adverse events reported in more than 3% of patients overall (Treated set) **7**

Table S9: Serious treatment-emergent adverse events **8**

Table S10 Complementary analyses comparing the success rate at 18 months with acoziborole in late-stage HAT patients with historical NECT data **8**

Table S11: Drug-related TEAEs by SOC and PT (Treated set) **9**

Table S12: HAT signs and symptoms by visit **9**

Figure S1: Derivation algorithm for patients with late-stage g-HAT at Month 18 (primary efficacy endpoint) **10**

Figure 2: Derivation algorithm for patients with late-stage g-HAT at Month 12 (secondary efficacy endpoint) **12**

Figure S3: HAT signs and symptoms by visit **14**

Figure 4 Yardstick **15**

Figure 5 Kaplan Meier analysis **16**

References **17**

Table S1: Schedule of assessments

Protocol procedures and forms to be completed	Pre-screening and screening	Baseline	Observation period until End-of-hospitalization Visit (EoH)															Out-patient follow-up period	
Timing →	D-15 to D-1	D-4 to D-1	D1	D2	D3	D4	D5	D6	D7	D8	D9	D10	D11	D12	D13	D14	D15 (EoH)	M3, M6, M12 & M18	Unscheduled Visit[1]
Diagnosis, i.e., detection of parasite in blood and/or lymph	x												x					x (M6, M12 & M18 only)	x
Lumbar puncture (parasite and WBC in CSF)	x												x					x (M6, M12 & M18 only)	x
Informed consent	x	Check																	
Pre-treatment of helminthiasis (+3-day recovery period)	x																		
Rapid diagnostic test and/or thick blood smear for malaria	x																		
Pre-treatment of malaria as needed (+3-day recovery period)	x																		
Karnofsky index	x	Check																x	x
Urinary pregnancy test (only for women)	x	x											x					x (up to M6)	x
Inclusion/exclusion criteria	x	Check																	
Demographic data	x																		
Geographical data	x																		
Medical history, incl. symptoms, severity, and time since start of disease	x																		
Weight, height, and BMI	x	x					x						x				x	x	x
Vital signs[2]	x	x					x						x				x	x	x
Clinical examination[3]		x					x						x				x	x	x
Physical examination		x					x						x				x	x	x

**Protocol procedures and forms to be completed**	**Pre-screening and screening**	**Baseline**	**Observation period until End-of-hospitalization Visit (EoH)**															**Out-patient follow-up period**	
Timing →	D-15 to D-1	D-4 to D-1	D1	D2	D3	D4	D5	D6	D7	D8	D9	D10	D11	D12	D13	D14	D15 (EoH)	M3, M6, M12 & M18	Unscheduled Visit[1]
Neurological examination		x					x						x				x	x	x
Hematology/biochemistry	x	x*					x						x					x	x
Thyroid function tests		x											x					x	x
ECG (2 duplicates)[4]			x																
ECG triplicate recording[5]			x	x	x	x	x						x						
Administration of acoziborole			x																
Collection of AEs[6]	x	x	x	x	x	x	x	x	x	x	x	x	x	x	x	x	x	x	x
Collection of concomitant medication	x	x	x	x	x	x	x	x	x	x	x	x	x	x	x	x	x	x (up to M6)	x
Blood sampling for PK analyses[7]			x	x	x	x	x						x					x (up to M6)	
CSF sampling for PK analyses[8]													x						

AE=Adverse event. BMI=Body mass index. CSF=Cerebrospinal fluid. D=Day. ECG=Electrocardiogram. EoH=End-of-hospitalisation. H=Hour. HAT=Human African trypanosomiasis. Incl.=Including. M=Month. PK=Pharmacokinetics. WBC=White blood cell count. *If the tests were performed prior to D-4 in the pre-screening/screening period or if the findings included abnormalities requiring repeat tests. [1] If relapse of the disease was suspected or if the patient did not feel well, s/he was to attend an unscheduled visit during which assessments, including additional safety assessments (at the Investigator's discretion) were to be performed, as well as investigation of concomitant disease. [2]Body temperature, blood pressure, heart rate, respiratory rate, and general status. [3]Row for "clinical examination", corresponding to the assessment of various signs and symptoms of HAT, added for completeness. [4] Prior to administration of acoziborole, D1H0: 2 x 2 ECG. [5]D1H4, D1H9, D1H24 (D2), D1H48 (D3), D1H72 (D4), D1H96 (D5), D1H240 (D11). [6]Adverse events were to be collected up to the M6 visit. Serious AEs were to be collected up to the M18 visit. [7]D1H0, D1H4; D1H9; D1H24 (D2); D1H48 (D3); D1H72 (D4); D1H96 (D5); D1H240 (D11); D90 (M3); D180 (M6). [8]Pharmacokinetics analyses performed on CSF sample collected for detection of parasites.

Table S2: Study objectives

**General objective**<br>To assess the efficacy and safety of a single oral dose of acoziborole administered to patients in the fasting state with g-HAT.
**Primary objective**<br>To estimate the success rate at 18 months of follow-up with acoziborole, administered as a single 960 mg oral dose to patients in the fasting state with late-stage g-HAT.<br><br>An estimate of the success rate observed with NECT in patients with late-stage g-HAT, based on historical data, was provided as a yardstick.
**Secondary objectives**<br>• To estimate the success rate at 12 months in late-stage patients;<br>• To estimate the time course of the failure rate in patients with late-stage g HAT;<br>• To assess the safety profile of a single dose of acoziborole in patients with g HAT using historical data on NECT as a yardstick;<br>• To establish the relationship between, on the one hand, concentrations of acoziborole in the blood and the CSF and, on the other hand, the efficacy and safety of acoziborole.<br>A cohort of patients 15 years of age or older with early- or intermediate-stage g-HAT was to be enrolled following a futility analysis, provided that it did not show futility and that the safety review showed no concerns. The overall objective was to assess the efficacy of acoziborole in this cohort and to enrich the safety database at the time of regulatory filing:<br>• To estimate the success rate with acoziborole in this cohort, using pentamidine as a yardstick, in order to verify the underlying hypothesis according to which, in HAT, a treatment able to clear the parasite from the CSF also clears it from the other compartments;<br>• To assess the safety profile in this cohort and in the overall population;<br>• To assess the safety of acoziborole in patients with early- and intermediate stage HAT to determine whether its safety profile is comparable to the historical safety profile of pentamidine.

CSF=Cerebrospinal fluid. g-HAT=Human African trypanosomiasis caused by *Trypanosoma brucei gambiense*. NECT=Nifurtimox-eflornithine combination therapy.

Table S3: Criteria for evaluation

**Efficacy**<br>*Primary efficacy endpoint*<br>Outcome (success or failure) of treatment at Month 18 for patients with late stage HAT according to the adapted WHO criteria (death considered a failure, regardless of the cause).
Secondary efficacy endpoints<br>• Response (success or failure) at Month 12 in patients with late-stage HAT;<br>• Response (success or failure) at Month 6 in patients with late-stage HAT;<br>• Response (success or failure) at Month 18 in patients with early and intermediate-stage HAT (specific algorithm for success determination);<br>• Response (success or failure) at Month 12 in patients with early and intermediate-stage HAT (specific algorithm for success determination);<br>• Response (success or failure) at Month 6 in patients with early and intermediate-stage HAT (specific algorithm for success determination);<br>• Time to proven and definitive failure in patients with late-stage HAT.
Additional efficacy endpoint<br>• Overall success rate at Month 18 (irrespective of the stage of HAT and the algorithm used for determining success).
**Safety**<br>Evaluation of TEAEs, standard haematology and biochemistry, ECG, vital signs, clinical signs and symptoms of HAT, and physical and neurological examination.
**Pharmacokinetics**<br>• Concentrations of acoziborole in whole blood and CSF, and population PK parameters, i.e., clearance, area under the curve and half-life;<br>• The correlation between exposure and efficacy/safety of acoziborole was also explored.
**Electrocardiograms**<br>ECGs were recorded at various time points to assess the QT interval/Fridericia's corrected QT, as well as other ECG parameters, and variations over time.

CSF=Cerebrospinal fluid. ECG=Electrocardiogram. g-HAT=Human African trypanosomiasis caused by *Trypanosoma brucei gambiense*. NECT=Nifurtimox-eflornithine combination therapy. PK=Pharmacokinetics. TEAE=Treatment-emergent adverse event. WHO=World Health Organization.

Table S4: Analysis sets

Primary efficacy set
mITT set:* All treated patients, excluding those who fled the region due to armed conflict, or natural disaster, or force majeure and for whom no failure was detected early* and no data were available at Month 12 and Month 18**<br>* Parasite, need for rescue medication, death, or more than 50 WBC/µL in CSF at Month 6.<br>** Due to armed conflict, natural disaster, or force majeure affecting the site.
**Other sets**
Screened HAT-positive set: All HAT positive patients who signed the informed consent. To be noted that after the first Data Safety Monitoring Board meeting, recruitment of early- and intermediate stage HAT patients was authorized, triggering a modification of the initial screened set.
Treated set†: All patients who received at least one tablet of acoziborole
EP set: All mITT patients, excluding those who were lost to follow-up (except if already a failure before being lost to follow-up); who had no post treatment lumbar puncture (but were not a failure); who died for reasons clearly unrelated to efficacy, safety, or disease evolution; or who withdrew consent before the Month 6 visit
PP set†: all mITT patients with no major protocol deviations

CSF=Cerebrospinal fluid. EP=Evaluable population. HAT=Human African trypanosomiasis. mITT=Modified intention-to-treat. PP=Per-protocol. WBC=White blood cell count. *Although no intention-to-treat (ITT) set was formally defined, the ITT set would be identical to the mITT set in this study since no patients were excluded from this analysis set. †In this study the mITT set was also identical to the Treated set and the PP set.

Table S5: Statistical methods

Primary efficacy variable
Primary analysis: An estimate of the success rate at Month 18 using the derivation algorithm for success, and the 95% Jeffreys CI of the estimate were provided.
Sensitivity analyses: The primary analysis was repeated on the PP, EP and Treated sets, and on the mITT set using different imputation methods (fair case, best case, and observed case).<br>note: the mITT set was identical to both the PP and Treated sets.
Complementary analyses: These compared the success rate with acoziborole at 18 months estimated by the primary analysis with historical success rates with NECT (i study by Priotto et al[1]; ii study by Mesu et al[2]; iii NECT-Field study[3]) and fexinidazole (study by Mesu et al[2]) as follows:<br>• The natural logarithm of WBC in CSF at baseline was used as covariate in a binary logistic model, ie, outcome at 18 months (success versus [vs.] failure) according to treatment (NECT/fexinidazole vs. acoziborole) and natural logarithm of baseline WBC in CSF;<br>• The analysis set was split in strata according to thresholds based on baseline WBC in CSF (<100 cells/µL, ≥100 cells/µL and <400 cells/µL, and ≥400 cells/µL). A Cochran–Mantel–Haenszel test was then performed for the comparison, ie, outcome at 18 months (success vs. failure) according to treatment (NECT/fexinidazole vs. acoziborole), adjusted for the strata;<br>• The natural logarithm of WBC in CSF at baseline, age and gender were used as covariates in a binary logistic model to obtain a propensity score. The analysis of the outcome at 18 months was then adjusted using propensity score.
**Secondary efficacy variables**
• Success rate at Month 12 and Month 6 in late-stage patients was analysed as described for the primary endpoint (including sensitivity analyses) but using specific derivation algorithms for the respective time points;<br>• Success rate at Month 18, Month 12, and Month 6 in early and intermediate stage patients was analysed as described for the primary and secondary outcome endpoints in late-stage patients (including sensitivity analyses) but using specific derivation algorithms for that cohort. The yardstick for this cohort was pentamidine;<br>• Time to proven and definitive failure was analysed according to cumulative failure rate using a Kaplan Meier approach;<br>• Changes in the rate of favourable outcomes over time were analysed by cohort using a logistic mixed model for repeated measures. A Cochran-Armitage trend test was used to assess the relationship between success rate at Month 18 (and Month 12) and ordered class of disease progress (ie, early- and late stage);<br>• A Cochran-Armitage trend test was used to assess the relationship between success rate at Month 18 (and Month 12) and subgroups defined according to stage of disease.
**Additional efficacy variable**<br>Overall success rate at Month 18 (irrespective of stage of disease) was calculated, along with the 95% Jeffreys CI.

CI=Confidence interval. CSF=Cerebrospinal fluid. EP=Evaluable population. NECT=Nifurtimox-eflornithine combination therapy. PP=Per-protocol. WBC=White blood cell count. *Although no intention-to-treat (ITT) set was formally defined, the ITT set would be identical to the mITT set in this study since no patients were excluded from this analysis set. †In this study the mITT set was also identical to the Treated set and the PP set.

Table S6: Historical data with NECT in late-stage g-HAT patients: determination of yardstick

Study	Success rate at 18 months for NECT ITT and 95% Jeffreys CI	Comments
Mesu 2018[2]	Success rate: 124/130=95.4%<br>95% CI: 90.7%; 98.1%	Pivotal Fexinidazole study<br>mITT:<br>Success rate: 124/127 = 97.6% (RZD trial)<br>95% Jeffreys CI: 93.8%-99.3%<br>Reason: 3 patients fleeing the region due to armed conflict
Priotto 2006[4]	Success rate: 16/17=94.1%<br>95% CI: 75.6%; 99.4%	RZD trial
Checchi 2007[5]	Success rate: 29/31=93.5%<br>95% CI: 80.9%; 98.6%	
Priotto 2009[1]	Success rate: 135/143=94.4%<br>95% CI: 89.7%; 97.3%	Success rate: 138/143=96.5%<br>95% Jeffreys CI: 92.5%; 98.7%<br>Note: exclusion of 3 deaths not related to treatment (RZD trial)
Kansiime 2018[6]	Success rate: 50/55=90.9%<br>95% CI: 81.2%; 96.4%	RZD trial
NECT-Field[3]	Success rate: 582/613=94.9%<br>95% CI: 93.0%; 96.5%	Evaluations were not always done at Month 18 (one-arm field study with loose monitoring), 49 patients LTFU at Month 18 were not necessarily counted as failure
**Total**	**Success rate: 354/376=94.1%**<br>**95% CI: 91.4%; 96.2%**	**Overall success rate excluding NECT-field.**

CI=Confidence interval. ITT=Intention-to-treat. mITT=Modified intention-to-treat. LTFU=Lost to follow-up. NECT=Nifurtimox-eflornithine combination therapy. RZD=Randomised.

Table S7: Success rates at 18, 12 and 6 months (mITT set)

	Early/intermediate-stage N=41	Late-stage N=167	Overall N=208
**18 months**			
Treatment success	41 (100·0%) [94·1; 100·0]	159 (95·2%) [91·2; 97·7]	200 (96·2%) [92·9; 98·2]
Treatment failure	-	8 (4·8%) [2·3; 8·8]	8 (3·8%) [1·8; 7·1]
**12 months**			
Treatment success	41 (100·0%) [94·1; 100·0]	160 (95·8%) [91·9; 98·1]	-
Treatment failure	-	7 (4·2%) [1·9; 8·1]	-
**6 months**			
Treatment success	41 (100·0%) [94·1; 100·0]	158 (94·6%) [90·4; 97·3]	-
Treatment failure	-	9 (5·4%) [2·7; 9·6]	-

Data are presented as n (%) [95% CI]. CI=Confidence interval. mITT=modified intention-to-treat.

Table S8: Summary of treatment-emergent adverse events reported in more than 3% of patients overall (Treated set)

	Early/intermediate-stage (n=41)	Late-stage (n=167)	Overall (n=208)
All TEAEs	28 (68·3%: 51.9; 81.9) [99]	127 (76·0%: 68.8; 82.3) [501]	155 (74·5%; 68.0; 80.3) [600]
Injury, poisoning and procedural complications	17 (41·5%: 26.3; 57.9) [31]	56 (33·5%: 26.4; 41.2) [72]	73 (35·1%; 28.6; 42.0) [103]
Procedural pain	13 (31·7%:18.1; 48.1) [17]	39 (23·4%: 17.2; 30.5) [46]	52 (25·0%: 19.3; 31.5) [63]
Procedural headache	11 (26·8%: 14.2; 42.9) [13]	21 (12·6%: 8.0; 18.6) [22]	32 (15·4%: 10.8; 21.0) [35]
Nervous system disorders	14 (34·1%: 20.1; 50.6) [16]	51 (30·5%: 23.7; 38.1) [68]	65 (31·3%: 25.0; 38.0) [84]
Headache	12 (29·3%: 16.1; 45.5) [14]	39 (23·4%: 17.2; 30.5) [49]	51 (24·5%: 18.8; 31.0) [63]
Infections and infestations	8 (19·5%: 8.8; 34.9) [12]	57 (34·1%: 25.5; 39.8) [103]	65 (31·3%: 25.0; 38.0) [115]
Malaria	5 (12·2%: 4.1; 26.2) [5]	24 (14·4%: 9.4; 20.6) [29]	29 (13·9%: 9.5; 19.4) [34]
Urinary tract infection	1 (2·4%: 0.1; 12.9) [1]	7 (4·2%; 1.7; 8.4) [10]	8 (3·8%: 1.7; 7.4) [11]
Diarrhoea infectious	2 (4·9%: 0.6; 16.5) [2]	5 (3·0%: 1.0; 6.8) [6]	7 (3·4%: 1.4; 6.8) [8]
General disorders and administration site<br>conditions	9 (22·0%: 10.6; 37.6) [9]	40 (24·0%: 17.7; 31.2) [55]	49 (23·6%: 17.8; 29.9) [64]
Pyrexia	4 (9·8%: 2.7; 23.1) [4]	27 (16·2%: 10.9; 22.6) [30]	31 (14·9%: 10.4; 20.5) [34]
Asthenia	5 (12·2%; 4.1; 26.2) [5]	13 (7·8%: 4.2; 12.9) [14]	18 (8·7%: 5.2; 13.3) [19]
Gastrointestinal disorders	2 (4·9%: 0.6; 16.5) [2]	32 (19·2%: 13.5; 26.0) [40]	34 (16·3%: 11.6; 22.1) [42]
Vomiting	0 (0·0%: 0.0; 8.6) [0]	13 (7·8%: 4.2; 12.9) [14]	13 (6·3%: 3.4; 10.5) [14]
Abdominal pain	0 (0·0%: 0.0; 8.6) [0]	8 (4·8%: 2.1; 9.2) [9]	8 (3·8%: 1.7; 7.4) [9]
Investigations	5 (12·2%: 4.1; 26.2) [5]	27 (16·2%: 10.9; 22.6) [31]	32 (15·4%: 10.8; 21.0) [36]
Weight increased	1 (2·4%; 0.1; 12.9) [1]	19 (11·4%: 7.0; 17.2) [19]	20 (9·6%: 6.0; 14.5) [20]
Psychiatric disorders	2 (4·9%:0.6; 16.5) [2]	28 (16·8%: 11.4; 23.3) [40]	30 (14·4%: 9.9; 20.0) [42]
Insomnia	2 (4·9%: 0.6; 16.5) [2]	13 (7·8%: 4.2; 12.9) [16]	15 (7·2%: 4.1; 11.6) [18]
Musculoskeletal and connective tissue disorders	6 (14·6%: 5.6; 29.2) [6]	20 (12·0%: 7.5; 17.9) [23]	26 (12·5%: 7.9; 17.2) [29]
Back pain	3 (7·3%:1.5; 19.9) [3]	10 (6·0%: 2.9; 10.7) [11]	13 (6·3%: 3.4; 10.5) [14]
Skin and subcutaneous tissue disorders	3 (7·3%: 1.5; 19.9) [4]	14 (8·4%: 4.7; 13.7) [17]	17 (8·2%: 4.8; 12.8) [21]
Pruritus	1 (2·4%: 0.1; 12.9) [1]	7 (4·2%: 1.7; 8.4) [7]	8 (3·8%: 1.7; 7.4) [8]
Metabolism and nutrition disorders	4 (9·8%: 2.7; 23.1) [4]	12 (7·2%:3.8; 12.2) [12]	16 (7·7%: 4.5; 12.2) [16]
Respiratory, thoracic and mediastinal disorders	1 (2·4%: 0.1; 12.9) [1]	9 (5·4%: 2.5; 10.0) [9]	10 (4·8%: 2.3; 8.7) [10]
Blood and lymphatic system disorders	1 (2·4%: 0.1; 12.9) [1]	7 (4·2%: 1.7; 8.4) [7]	8 (3·8%: 1.7; 7.4) [8]
Anaemia	1 (2·4%; 0.1; 12.9) [1]	6 (3·6%: 1.3; 7.7) [6]	7 (3·4%: 1.4; 6.8) [7]
Cardiac disorders	1 (2·4%: 0.1; 12.9) [1]	7 (4·2%: 1.7; 8.4) [8]	8 (3·8%: 1.7; 7.4) [9]

Data are presented as n (% and 95% C.I.) [number of events]. System organ classes are not presented if no event by preferred term is reported in a higher percentage of patients than the cut-off. TEAE=treatment-emergent adverse event.

Table S9: Serious treatment-emergent adverse events

SOC<br>PT	Early/intermediate-stage (N=41)	Late-stage (N=167)	Overall (N=208)
Any serious TEAE	3 (7·3%: 1.5; 19.9) [4]	18 (10·8%: 28.5; 60.3) [23]	21 (10·1%: 6.4; 15.0) [27]
Infections and Infestations	1 (2·4%: 0.1; 12.9) [1]	8 (4·8%: 2.1; 9.2) [9]	9 (4·3%: 2.0; 8.1) [10]
Malaria	0 (0·0%: 0.0; 8.6) [0]	2 (1·2%: 0.1; 4.3) [2]	2 (1·0%: 0.1; 3.4) [2]
Appendicitis	0 (0·0%: 0.0; 8.6) [0]	1 (0·6%: 0.0; 3.3) [1]	1 (0·5%: 0.0; 2.6) [1]
Extrapulmonary tuberculosis	0 (0·0%: 0.0; 8.6) [0]	1 (0·6%: 0.0; 3.3) [1]	1 (0·5%: 0.0; 2.6) [1]
Gastroenteritis	0 (0·0%: 0.0; 8.6) [0]	1 (0·6%: 0.0; 3.3) [2]	1 (0·5%: 0.0; 2.6) [2]
Puerperal infection	1 (2·4%: 0.1; 12.9) [1]	0 (0·0%: 0.0; 2.2) [0]	1 (0·5%: 0.0; 2.6) [1]
Systemic infection	0 (0·0%: 0.0; 8.6) [0]	1 (0·6%: 0.0; 3.3) [1]	1 (0·5%: 0.0; 2.6) [1]
Typhoid fever	0 (0·0%: 0.0; 8.6) [0]	1 (0·6%: 0.0; 3.3) [1]	1 (0·5%: 0.0; 2.6) [1]
Upper respiratory tract infection	0 (0·0%: 0.0; 8.6) [0]	1 (0·6%: 0.0; 3.3) [1]	1 (0·5%: 0.0; 2.6) [1]
Psychiatric Disorders	0 (0·0%: 0.0; 8.6) [0]	6 (3·6%: 1.3; 7.7) [7]	6 (2·9%: 1.1; 6.2) [7]
Acute psychosis	0 (0·0%: 0.0; 8.6) [0]	1 (0·6%: 0.0; 3.3) [1]	1 (0·5%: 0.0; 2.6) [1]
Bipolar I disorder	0 (0·0%: 0.0; 8.6) [0]	1 (0·6%: 0.0; 3.3) [1]	1 (0·5%: 0.0; 2.6) [1]
Brief psychotic disorder with marked stressors	0 (0·0%: 0.0; 8.6) [0]	1 (0·6%: 0.0; 3.3) [1]	1 (0·5%: 0.0; 2.6) [1]
Major depression	0 (0·0%: 0.0; 8.6) [0]	1 (0·6%: 0.0; 3.3) [1]	1 (0·5%: 0.0; 2.6) [1]
Mania	0 (0·0%: 0.0; 8.6) [0]	1 (0·6%: 0.0; 3.3) [1]	1 (0·5%: 0.0; 2.6) [1]
Organic brain syndrome	0 (0·0%: 0.0; 8.6) [0]	1 (0·6%: 0.0; 3.3) [1]	1 (0·5%: 0.0; 2.6) [1]
Suicide attempt	0 (0·0%: 0.0; 8.6) [0]	1 (0·6%: 0.0; 3.3) [1]	1 (0·5%: 0.0; 2.6) [1]
Gastrointestinal Disorders	1 (2·4%: 0.1; 12.9) [1]	1 (0·6%: 0.0; 3.3) [1]	2 (1·0%: 0.1; 3.4) [2]
Abdominal adhesions	1 (2·4%: 0.1; 12.9) [1]	0 (0·0%: 0.0; 3.3) [0]	1 (0·5%: 0.0; 2.6) [1]
Inguinal hernia	0 (0·0%: 0.0; 8.6) [0]	1 (0·6%: 0.0; 3.3) [1]	1 (0·5%: 0.0; 2.6) [1]
Nervous System Disorders	0 (0·0%: 0.0; 8.6) [0]	2 (1·2%: 0.1; 4.3) [2]	2 (1·0%: 0.1; 3.4) [2]
Guillain-Barre syndrome	0 (0·0%: 0.0; 8.6) [0]	1 (0·6%: 0.0; 3.3) [1]	1 (0·5%: 0.0; 2.6) [1]
Intracranial pressure increased	0 (0·0%: 0.0; 8.6) [0]	1 (0·6%: 0.0; 3.3) [1]	1 (0·5%: 0.0; 2.6) [1]
Respiratory, Thoracic and Mediastinal Disorders	0 (0·0%: 0.0; 8.6) [0]	2 (1·2%: 0.1; 4.3) [2]	2 (1·0%: 0.1; 3.4) [2]
Acute pulmonary oedema	0 (0·0%: 0.0; 8.6) [0]	1 (0·6%: 0.0; 3.3) [1]	1 (0·5%: 0.0; 2.6) [1]
Choking	0 (0·0%: 0.0; 8.6) [0]	1 (0·6%: 0.0; 3.3) [1]	1 (0·5%: 0.0; 2.6) [1]
Injury, Poisoning and Procedural Complications	0 (0·0%: 0.0; 8.6) [0]	1 (0·6%: 0.0; 3.3) [1]	1 (0·5%: 0.0; 2.6) [1]
Poisoning	0 (0·0%: 0.0; 8.6) [0]	1 (0·6%: 0.0; 3.3) [1]	1 (0·5%: 0.0; 2.6) [1]
Pregnancy, Puerperium and Perinatal Conditions	1 (2·4%: 0.1; 12.9) [2]	0 (0·0%: 0.0; 2.2) [0]	1 (0·5%: 0.0; 2.6) [2]
Postpartum haemorrhage	1 (2·4%: 0.1; 12.9) [1]	0 (0·0%: 0.0; 2.2) [0]	1 (0·5%: 0.0; 2.6) [1]
Umbilical cord prolapse	1 (2·4%: 0.1; 12.9) [1]	0 (0·0%: 0.0; 2.2) [0]	1 (0·5%: 0.0; 2.6) [1]
Reproductive System and Breast Disorders	0 (0·0%: 0.0; 8.6) [0]	1 (0·6%: 0.0; 3.3) [1]	1 (0·5%: 0.0; 2.6) [1]
Ovarian cyst	0 (0·0%: 0.0; 8.6) [0]	1 (0·6%: 0.0; 3.3) [1]	1 (0·5%: 0.0; 2.6) [1]

Data are presented as n (% and 95% C.I.) [number of events]. PT=Preferred term. SOC=System organ class. TEAE=Treatment-emergent adverse event.

Table S10: Complementary analyses comparing the success rate at 18 months with acoziborole in late-stage HAT patients with historical NECT data

	Acoziborole vs NECT (Priotto et al, 2009)	Acoziborole vs. NECT (Mesu et al, 2018)	Acoziborole vs. NECT (NECT-Field)
Comparison adjusted for baseline CSF WBC count	0·567 (0·168; 1·912)	-*	0·873 (0·390; 1·957)
Comparison adjusted for the natural logarithm of baseline CSF WBC count	0·5319 (0·156; 1·759)	1·943 (0·542; 9·094)	0·824 (0·344; 1·761)
Comparison adjusted by propensity score	0·5354 (0·159; 1·766)	2·04 (0·563; 9·64)	0·814 (0·340; 1·738)

Data are presented as odds ratio (95% CI). *The odds ratio across strata was not homogenous.

Table S11: Drug-related TEAEs by SOC and PT (Treated set)

**SOC**<br>**PT**	**Early/intermediate-stage (N=41)**	**Late-stage (N=167)**	**Overall (N=208)**
Any drug-related TEAE	2 (4.9%) [2]	27 (16.2%) [36]	29 (13.9%) [38]
General Disorders and Administration Site Conditions	0 (0·0%) [0]	15 (9.0%) [17]	15 (7.2%) [17]
Pyrexia	0 (0·0%) [0]	10 (6.0%) [10]	10 (4.8%) [10]
Asthenia	0 (0·0%) [0]	6 (3·6%) [6]	6 (2.9%) [6]
Chills	0 (0·0%) [0]	1 (0·6%) [1]	1 (0·5%) [1]
Nervous System Disorders	1 (2.4%) [1]	7 (4·2%) [7]	8 (3.8%) [8]
Tremor	0 (0·0%) [0]	3 (1.8%) [3]	3 (1.4%) [3]
Dyskinesia	1 (2.4%) [1]	1 (0·6%) [1]	2 (1·0%) [2]
Headache	0 (0·0%) [0]	2 (1.2%) [2]	2 (1·0%) [2]
Dizziness	0 (0·0%) [0]	1 (0·6%) [1]	1 (0·5%) [1]
Gastrointestinal Disorders	0 (0·0%) [0]	4 (2.4%) [5]	4 (1.9%) [5]
Abdominal pain	0 (0·0%) [0]	2 (1.2%) [3]	2 (1.0%) [3]
Nausea	0 (0·0%) [0]	1 (0·6%) [1]	1 (0·5%) [1]
Vomiting	0 (0·0%) [0]	1 (0·6%) [1]	1 (0·5%) [1]
Metabolism and Nutrition Disorders	1 (2.4%) [1]	3 (1.8%) [3]	4 (1.9%) [4]
Decreased appetite	1 (2.4%) [1]	3 (1.8%) [3]	4 (1.9%) [4]
Eye Disorders	0 (0·0%) [0]	1 (0·6%) [1]	1 (0·5%) [1]
Vision blurred	0 (0·0%) [0]	1 (0·6%) [1]	1 (0·5%) [1]
Investigations	0 (0·0%) [0]	1 (0·6%) [1]	1 (0·5%) [1]
Electrocardiogram T-wave inversion	0 (0·0%) [0]	1 (0·6%) [1]	1 (0·5%) [1]
Renal and Urinary Disorders	0 (0·0%) [0]	1 (0·6%) [1]	1 (0·5%) [1]
Chromaturia	0 (0·0%) [0]	1 (0·6%) [1]	1 (0·5%) [1]
Skin and Subcutaneous Tissue Disorders	0 (0·0%) [0]	1 (0·6%) [1]	1 (0·5%) [1]
Pruritus	0 (0·0%) [0]	1 (0·6%) [1]	1 (0·5%) [1]

Data are presented as n (%) [number of events]. PT=Preferred term. SOC=System organ class. TEAE=Treatment-emergent adverse event.

Table S12: Evolution of HAT signs and symptoms which were present in >20% of patients at baseline by visit

**Signs and Symptoms**	**Screening (N=208)**	**D15 (N=208)**	**M3 (N=205)**	**M6 (N=205)**	**M12 (N=203)**	**M18 (N=201)**
Headaches	62.5% (130)	4.3% (9)	2.0% (4)	2.4% (5)	0.0% (0)	0.0% (0)
Drowsiness	62.5% (130)0	10.6% (22)	0.5% (1)	0.0% (0)	0.0% (0)	0.0% (0)
Asthenia	51.4% (107)	5.8% (12)	2.0% (4)	2.0% (4)	0.0% (0)	0.0% (0)
Pruritus	51.4% (107)	22.6% (47)	11.2% (23)	6.8% (14)	0.0% (0)	0.0% (0)
Fever	44.7% (93)	0.5% (1)	2.4% (5)	2.0% (4)	0.0% (0)	0.0% (0)
Insomnia	37.0% (77)	7.7% (16)	5.9% (12)	2.0% (4)	0.0% (0)	0.0% (0)
Thinning/weight loss	37.0% (77)	1.0% (2)	2.0% (4)	1.5% (3)	0.0% (0)	0.0% (0)
Tremor	31.7% (66)	10.1% (21)	1.5% (3)	0.5% (1)	0.0% (0)	0.0% (0)
Gait disturbances	22.1% (46)	8.2% (17)	1.5% (3)	0.5% (1)	0.0% (0)	0.0% (0)

Data are presented as % (number of patients with signs and symptoms). D=day, M=month.

Figure S1: Derivation algorithm for patients with late-stage g-HAT at Month 18 (primary efficacy endpoint)

Patient is ⇨ 1: Dead (any reason of death and any time between drug intake and Month 18)

⇨ **Failure** (stop)

⇩

Alive

⇩

Patient requires rescue medication for HAT at Month 18 or required it before (between drug intake and Month 18)

⇨ YES ⇨ 2: (Definitive) **failure** (stop)

⇩

NO rescue medication so far

⇩

Evidence of trypanosomes in any body fluid between drug intake and Month 18

⇨ YES ⇨ 3: (Definitive) **failure** (stop)

⇩

NO observed trypanosomes

⇩

Patient lost to follow-up at Month 18 (no survival information at Month 18 and later)

⇨ YES ⇨ 4: **Failure** (stop)[*,†]

⇩

NO (The patient is not lost to follow-up)

⇩

Non-hemorrhagic lumbar puncture at Month 18 and WBC in CSF at Month 18 >20 cells/µL

⇨ YES ⇨ 5: **Failure** (stop)

⇩

NO (WBC in CSF at Month 18 ≤20 cells/µL or no reliable data concerning WBC in CSF at Month 18)

⇩

Non-hemorrhagic lumbar puncture at Month 18 and WBC in CSF ≤20 cells/µL

⇨ YES ⇨ 6: **Success** (stop)

⇩

NO (hemorrhagic CSF sample or no lumbar puncture at Month 18 for any reason)

⇩

No lumbar puncture at Month 18 or no reliable count of WBC in CSF at Month 18 but reliable number of WBC in CSF reported later (Month 24[‡] or other additional visit)

⇨ YES ⇨ **WBC in CSF >20 cells/µL** ⇨ 7: **Failure** (stop)

⇨ YES ⇨ **WBC in CSF ≤20 cells/µL** ⇨ 8: **Success** (stop)

⇩

NO (No lumbar puncture at Month 18 and no later reliable count of WBC in CSF)

⇩

Earlier (before Month 18) unfavorable outcome: WBC in CSF >50 cells/µL at Month 6 or WBC in CSF >20 cells/µL at Month 12 or increasing between Month 6 and Month 12[§]

YES ⇨ 9: **Failure** (stop)

⇩

NO (no information or no earlier unfavorable assessment)

⇩

Patient has clinical signs or symptoms at Month 18 evoking a failure

YES 10: **Failure** (stop)

⇩

NO (no clinical signs or symptoms evoking a relapse at Month 18)

⇩

WBC in CSF ≤20 cells/µL at Month 12 or in absence of lumbar puncture at Month 12 and for both cases WBC in CSF ≤50 cells/µL at Month 6 and no signs and symptoms at Month 18 evoking a relapse

YES 11: **Success** (stop)[†]

⇩

NO (at least one criterion not met)

⇩

Patient refused all post-treatment lumbar punctures

YES 12: **Failure** (stop)[*,†]

⇩

All other cases ⇨ 13: Failure

CSF=Cerebrospinal fluid. HAT=Human African trypanosomiasis. WBC=White blood cell count. *Was considered as a success for sensitivity analysis with the best case method. †Re-sampling was applied for sensitivity analysis with the fair case method. ‡No visit was planned at 24 months. §This was valid only if there was no visit at 18 months.

Figure S2: Derivation algorithm for patients with late-stage g-HAT at Month 12 (secondary efficacy endpoint)

Derivation algorithm at 12 months for stage 1 and intermediate stage	Derivation algorithm at 12 months for stage 2
**Patient is** ⇒ **1:** Dead (any reason of death and any time between drug intake and M12) ⇒ *(definitive)* ***Failure*** **(stop)**<br>⇓<br>Alive<br>⇓	**Patient is** ⇒ **1:** Dead (any reason of death and any time between drug intake and M12) ⇒ *(definitive)* ***Failure*** **(stop)**<br>⇓<br>Alive<br>⇓
**Patient requires rescue medication for HAT at M12 or before**<br>⇒ YES ⇒ 2: ***Failure*** **(stop)**<br>⇓<br>NO rescue medication so far<br>⇓	**Patient requires rescue medication for HAT at M12 or before**<br>⇒ YES ⇒ 2: ***Failure*** **(stop)**<br>⇓<br>NO rescue medication so far<br>⇓
**Evidence of trypanosomes in any body fluid at M12 or between drug intake and M12 visit**<br>⇒ YES ⇒ 3: (*definitive*) ***Failure*** **(stop)**<br>⇓<br>NO observed trypanosomes so far<br>⇓	**Evidence of trypanosomes in any body fluid at M12 or between drug intake and M12 visit**<br>⇒ YES ⇒ 3: (*definitive*) ***Failure*** **(stop)**<br>⇓<br>NO observed trypanosomes so far<br>⇓
**Patient Lost to follow-up at 12M** (no survival information at M12 and later)<br>⇒ YES ⇒ 4: ***Failure*** **(Stop) [a,b]**<br>⇓<br>NO (the patient is not lost to follow-up)<br>⇓	**Patient Lost to follow-up at 12M** (no survival information at M12 and later)<br>⇒ YES ⇒ 4: ***Failure*** **(Stop) [a,b]**<br>⇓<br>NO (the patient is not lost to follow-up)<br>⇓
**Non-hemorrhagic lumbar puncture at M12 and WBC in CSF >20 cells**<br>⇒YES ⇒ 5: ***Failure*** **(stop)**<br>⇓<br>NO (WBC in CSF at M12 ≤ 20 cells or no reliable count of WBC in CSF at M12)<br>⇓	**Non-hemorrhagic lumbar puncture at M12 and WBC in CSF at M12 ≥ 50 cells**<br>⇒ YES ⇒ **5: *Failure*(stop)**<br>⇓<br>NO (WBC in CSF at M12 < 50 cells or no reliable count of WBC in CSF at M12)<br>⇓
**Non-hemorrhagic lumbar puncture at M12 and WBC in CSF at M12 ≤ 20 cells**<br>⇒ YES ⇒ 6: ***Success*** **(Stop)**<br>⇓<br>NO (no reliable count of WBC in CSF at M12)<br>⇓	**Non-hemorrhagic lumbar puncture at M12 with WBC in CSF < 50 but > 20 and increase of WBC in CSF with respect to M6 or sign and symptoms evoking a failure**<br>⇒ YES ⇒ **6: *Failure* (stop)**<br>⇓<br>NO (patient did not meet at least one of the previous criteria at M12)<br>⇓
⇓<br>⇓<br>⇓<br>⇓	**Non-hemorrhagic lumbar puncture at M12 with WBC in CSF < 50 but > 20 cells and decrease of WBC in CSF with respect to M6 and no signs and symptoms evoking a failure (success at M18 if status is available)**<br>⇒ YES ⇒ 7: **Success (stop)**<br>⇓<br>NO (patient did not meet at least one of the previous criteria)<br>⇓
**No lumbar puncture at M12 or no reliable count of WBC in CSF at M12 but reliable number of WBC in CSF reported later** (M18 or other additional visit)	**No lumbar puncture at M12 or no reliable count of WBC in CSF at M12 but reliable number of WBC in CSF reported later** (M18 or other additional visit)

**Derivation algorithm at 12 months for stage 1 and intermediate stage**	**Derivation algorithm at 12 months for stage 2**
⇒ YES ⇒ **WBC in CSF > 20** ⇒ 7: ***Failure*** **(Stop)**<br>⇒ YES ⇒ **WBC in CSF ≤ 20 and no signs or symptoms evoking a relapse** ⇒ 8: ***Success*** **(Stop)**<br>⇓<br>NO (no later reliable count of WBC in CSF)<br>⇓<br>**No reliable WBC count in CSF at M12 and later and Failure at M18 for any reason**<br>⇒ YES ⇒ **9:** ***Failure*** at M12 **(Stop)**<br>⇓<br>NO<br>⇓<br>**No reliable count of WBC in CSF at M18 and M12 but WBC counts at M6 available and no signs and symptoms evoking a failure at M12 or M18**<br>⇒YES ⇒ **WBC in CSF at M6 ≤ 5 and smaller than WBC in CSF at EOH** ⇒ 10: ***Success*** **(stop) [b]**<br>⇒ NO ⇒ **WBC in CSF at M6 > 5 or larger than WBC in CSF at EOH** ⇒ *11:* ***Failure*** **(stop)**<br>⇓<br>NO (no lumbar puncture at M6, M12 and M18)<br>⇓<br>**Patient refused all post-treatment lumbar punctures but was met at M24 or later with no signs and symptoms evoking a relapse (normal activity)**<br>**⇒ YES ⇒ 12:** ***Success*** at M12 **[b]**<br>⇓<br>NO (Patient not met at M24 or later)<br>⇓<br>**Patient refused all post treatment lumbar punctures** ⇒ YES ⇒ 13: ***Failure*** **(Stop) [a,b]**	⇒ YES ⇒ **WBC in CSF > 20** ⇒ 8: ***Failure*** **(Stop)**<br>⇒ YES ⇒ **WBC in CSF ≤ 20** ⇒ 9: ***Success*** **(Stop)**<br>⇓<br>NO (no later reliable count of WBC in CSF)<br>⇓<br>**No reliable WBC count in CSF at M12 and later and Failure at M18 for any reason**<br>⇒ YES ⇒ 10: ***Failure*** at M12 **(Stop)**<br>⇓<br>NO<br>⇓<br>**No reliable count of WBC in CSF at M18 and M12 but WBC counts at M6 available and no sign and symptoms evoking relapse at M12 or M18**<br>YES ⇒ **WBC in CSF at M6 ≤ 20 cells** ⇒ ***11: Success (stop)*** **[b]**<br>NO ⇒ **WBC in CSF at M6 > 20 cells ⇒ 12:** ***Failure***<br>⇓<br>NO (no lumbar puncture at M6, M12 and M18)<br>⇓<br>**Patient refused all post-treatment lumbar punctures but was met at M24 or later with no signs and symptoms evoking a relapse (normal activity)**<br>**⇒ YES ⇒ 13:** ***Success*** at M12 **[b]**<br>⇓<br>NO (Patient not met at M24 or later)<br>⇓<br>**Patient refused all post treatment lumbar punctures** ⇒ YES ⇒ 14: ***Failure*** **(Stop) [a,b]**

For sensitivity analyses [a] will be considered as a success with the best case method and [b] re-sampling will be applied with the fair case method.

Figure S3: HAT signs and symptoms by visit

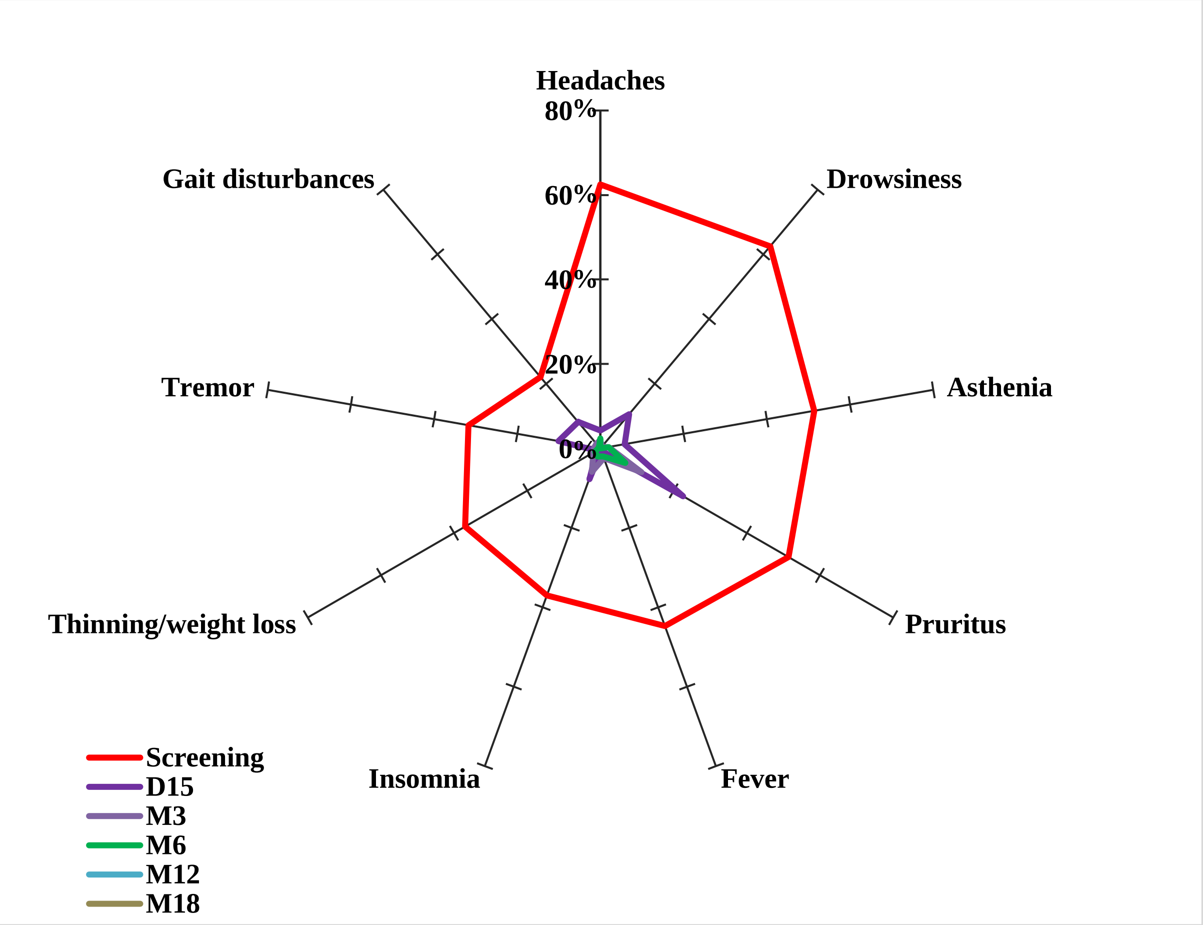

M = month, D = day of follow-up visit. The main clinical signs and symptoms were cleared after 3 months.

Figure S4: Success rate at Month 18 in late-stage HAT patients: acoziborole (mITT set) and the main (NECT) and secondary (fexinidazole) yardsticks

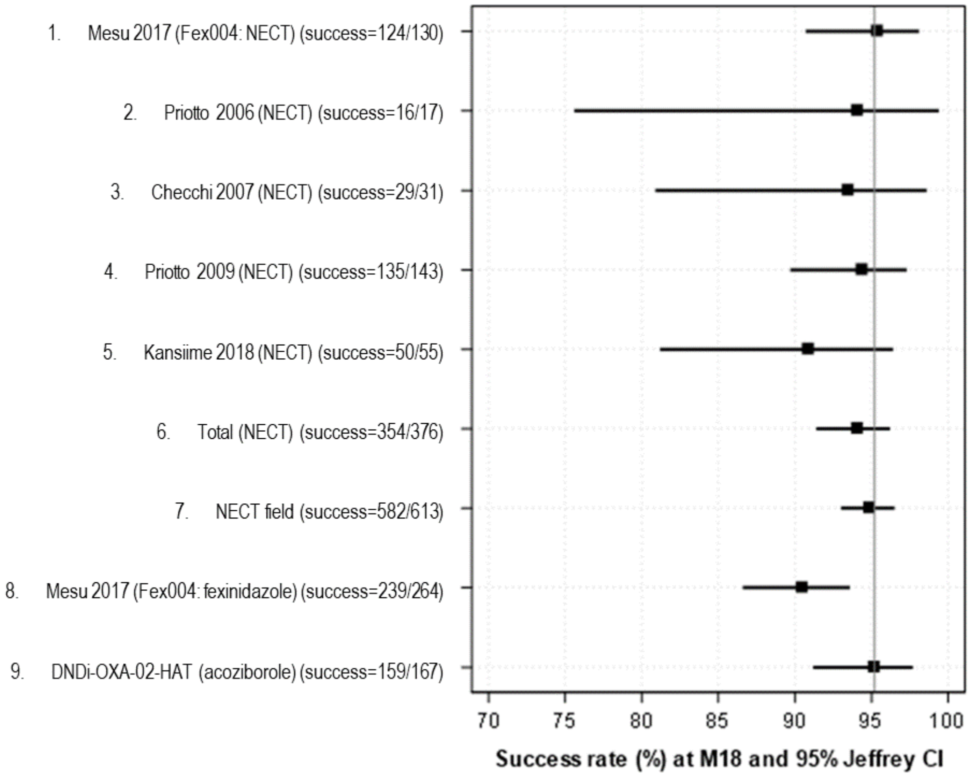

Note: results are presented in ITT populations instead of mITT because results on mITT are not available for all trials. Results on mITT with Fexinidazole were: success = 239 / 262 (91·2% - 95%, CI=87·3-94·2).

Figure S5: Proportion of proven failure free patients over time based on Kaplan-Meier analysis in patients with late-stage HAT (mITT set)

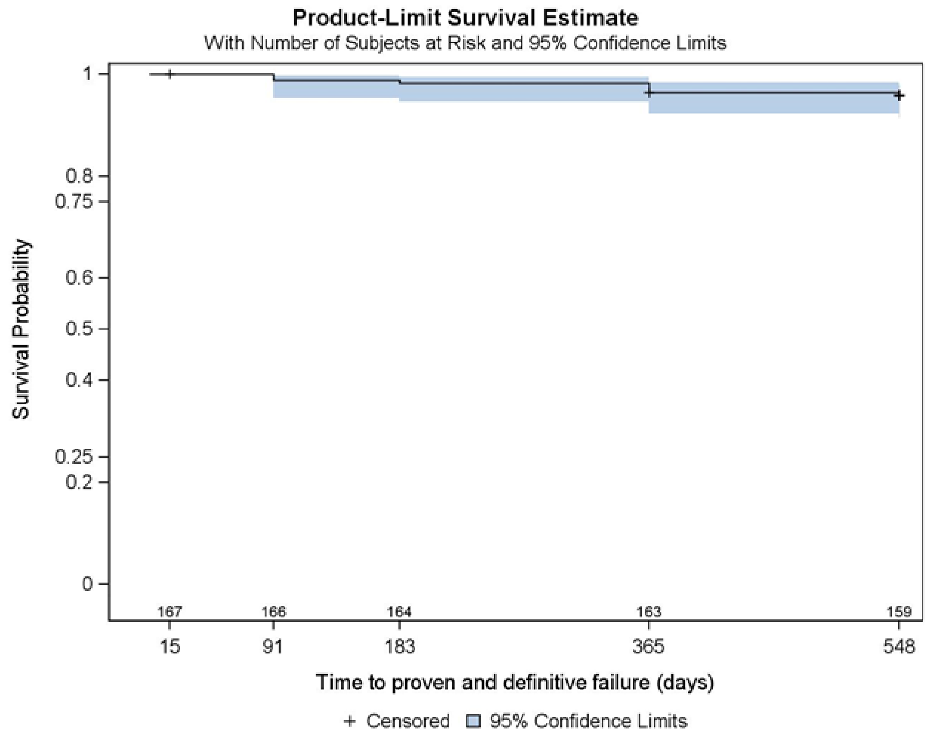

Events	Censored	Survival Probability	Survival time estimate (95% CI)
7	160	0.75	NE [NE;NE]
		0.50	NE [NE;NE]
		0.25	NE [NE;NE]

CI = confidence interval; HAT = human African trypanosomiasis; mITT = modified intention-to-treat; NE = not estimated. Time to 75%, 50% or 25% failure rate were not estimated due to the insufficient number of proven failures

References

1 Priotto G, Kasparian S, Mutombo W, et al. Nifurtimox-eflornithine combination therapy for second-stage African Trypanosoma brucei gambiense trypanosomiasis: a multicentre, randomised, phase III, non-inferiority trial. *Lancet* 2009; **374:** 56-64.

2 Mesu VK, Kalonji WM, Bardonneau C, et al. Oral fexinidazole for late-stage African Trypanosoma brucei gambiense trypanosomiasis: a pivotal multicentre, randomised, non-inferiority trial. *Lancet* 2018; **391:** 144-54.

3 Kuemmerle A, Schmid C, Bernhard S, et al. Effectiveness of Nifurtimox Eflornithine Combination Therapy (NECT) in T. b. gambiense Second Stage Sleeping Sickness Patients in the Democratic Republic of Congo: Report from a Field Study. *PloS Negl Trop Dis* 2021; **15:** e0009903.

4 Priotto G, Fogg C, Balasegaram M, et al. Three drug combinations for late-stage Trypanosoma brucei gambiense sleeping sickness: a randomized clinical trial in Uganda. *PLoS Clinical Trials* 2006; **1:** e39.

5 Checchi F, Piola P, Ayikoru H, Thomas F, Legros D, Priotto G. Nifurtimox plus eflornithine for late-stage sleeping sickness in Uganda: a case series. *PLoS Negl Trop Dis* 2007; **1:** e64.

6 Kansiime F, Adibaku S, Wamboga C, et al. A multicentre, randomised, non inferiority clinical trial comparing a nifurtimox eflornithine combination to standard eflornithine monotherapy for late stage Trypanosoma brucei gambiense human African trypanosomiasis in Uganda. *Parasit Vectors* 2018; **11:** 105.
